# Transcriptome analysis based on machine learning reveals a role for autoinflammatory genes of chronic nonbacterial osteomyelitis (CNO)

**DOI:** 10.1038/s41598-023-33759-y

**Published:** 2023-04-21

**Authors:** Zhuodong Fu, Xingkai Wang, Linxuan Zou, Zhe Zhang, Ming Lu, Junwei Zong, Shouyu Wang

**Affiliations:** 1 Department of Orthopedic Surgery, the First Affiliated Hospital of Dalian Medical University, 222 Zhongshan Road, Dalian, China; 2 Institute (College) of Integrative Medicine, Dalian Medical University, Dalian, Liaoning China; 3 Trauma and Tissue Repair Surgery Department, Dalian Municipal Central Hospital, Dalian, China

**Keywords:** Biological techniques, Genetics

## Abstract

Chronic nonbacterial osteomyelitis (CNO) is an autoinflammatory bone disorder. The origin and development of CNO involve many complex immune processes, resulting in delayed diagnosis and a lack of effective treatment. Although bioinformatics analysis has been utilized to seek key genes and pathways in CNO, only a few bioinformatics studies that focus on CNO pathogenesis and mechanisms have been reported. This study aimed to identify key biomarkers that could serve as early diagnostic or therapeutic markers for CNO. Two RNA-seq datasets (GSE133378 and GSE187429) were obtained from the Gene Expression Omnibus (GEO). Weighted gene coexpression network analysis (WGCNA) and differentially expressed gene (DEG) analysis were conducted to identify the genes associated with CNO. Then, the autoinflammatory genes most associated with CNO were identified based on the GeneCards database and a CNO prediction model, which was created by the LASSO machine learning algorithm. The accuracy of the model and effects of the autoinflammatory genes according to receiver operating characteristic (ROC) curves were verified in external datasets (GSE7014). Finally, we performed clustering analysis with ConsensusClusterPlus. In total, eighty CNO-related genes were identified and were significantly enriched in the biological processes regulation of actin filament organization, cell–cell junction organization and gamma-catenin binding. The main enriched pathways were adherens junctions, viral carcinogenesis and systemic lupus erythematosus. Two autoinflammatory genes with high expression in CNO samples were identified by combining an optimal machine learning algorithm (LASSO) with the GeneCards database. An external validation dataset (GSE187429) was utilized for ROC analysis of the prediction model and two genes, and the results indicated good efficiency. Then, based on consensus clustering analysis, we found that the expression of UTS2 and MPO differed between clusters. Finally, the ceRNA network of lncRNAs and the small molecule compounds targeting the two autoinflammatory genes were predicted. The identification of two autoinflammatory genes, the HCG18/has-mir-147a/UTS2/MPO axis and signalling pathways in this study can help us understand the molecular mechanism of CNO formation and provides candidate targets for the diagnosis and treatment of CNO.

## Introduction

Chronic nonbacterial osteomyelitis (CNO) is a rare autoinflammatory bone disease caused by abnormal activation of the innate immune system^1^. CNO has a wide range of clinical manifestations, from unifocal to multifocal lesions, and its more severe form is also known as chronic recurrent multifocal osteomyelitis (CRMO)^2^. CNO onset occurs mainly in children between the ages of 7 and 12, but the disease manifestations can continue into adulthood^3^. The incidence is estimated at approximately 0.4 to 2 cases per 100,000 children, and the prevalence is approximately twice as high in girls as in boys^3,4^.

Although the exact pathogenesis of CNO is unknown, several recent studies have suggested a multifactorial origin, including cytokine dysregulation, osteoclast activation, and genetic susceptibility. The levels of pro-inflammatory cytokines such as TNF-a and interleukin (IL)-6 have been reported to be increased in CNO patients, while those of anti-inflammatory cytokines, especially IL-10, are decreased^5^. In addition, abnormal regulation of the IL-1β axis may be associated with NLRP3 inflammatory vesicles in the pathogenesis of CNO^6^. These cytokines may cause osteoclast differentiation and activation by increasing the interaction of nuclear factor kappa-B (RANK) with its soluble ligand RANKL in osteoclasts, leading to bone destruction^7^. FBLIM1^8^, FGR^9^ and IL1RN^10^ are among the few genes that are known to intersect with the pathogenesis of CNO, but the exact mechanism is not yet known.

Although diagnostic criteria for CNO have been developed^11^, the increased sensitivity of MRI in identifying CNO has facilitated the diagnosis and long-term monitoring of patients with suspected or known CNO. However, the diagnosis of CNO is still an exclusionary diagnosis, and delayed diagnosis remains common, with an average delay of 2 years in children^12^. Although nonsteroidal anti-inflammatory drugs (NSAIDs) are used as first-line drugs for the treatment of CNO, there is no specific NSAID that is recommended, and the literature varies widely in the choice of drug^13^.

Given the current dilemmas of diagnostic uncertainty and vague treatment guidelines for CNO, there is an urgent need to find molecular biomarkers or therapeutic targets that can contribute to the early diagnosis of the disease. RNA-seq data has been widely utilized as a diagnostic tool to assess rare diseases with different pathophysiology^14–16^. In this study, we searched the Gene Expression Omnibus (GEO) database and obtained the GSE133378 and GSE187429 RNA-seq datasets, both containing gene expression data from CNO and normal samples. Moreover, we predicted long noncoding RNAs (lncRNAs), which are a class of ncRNA molecules involved in protein regulation, as upstream regulators of target genes to build ceRNA networks^17^. This helps us to reach the goal of exploring key biomarkers that could serve as early diagnostic or therapeutic markers for CNO.

## Materials and methods

### Dataset download and preprocessing

**Figure 1:**
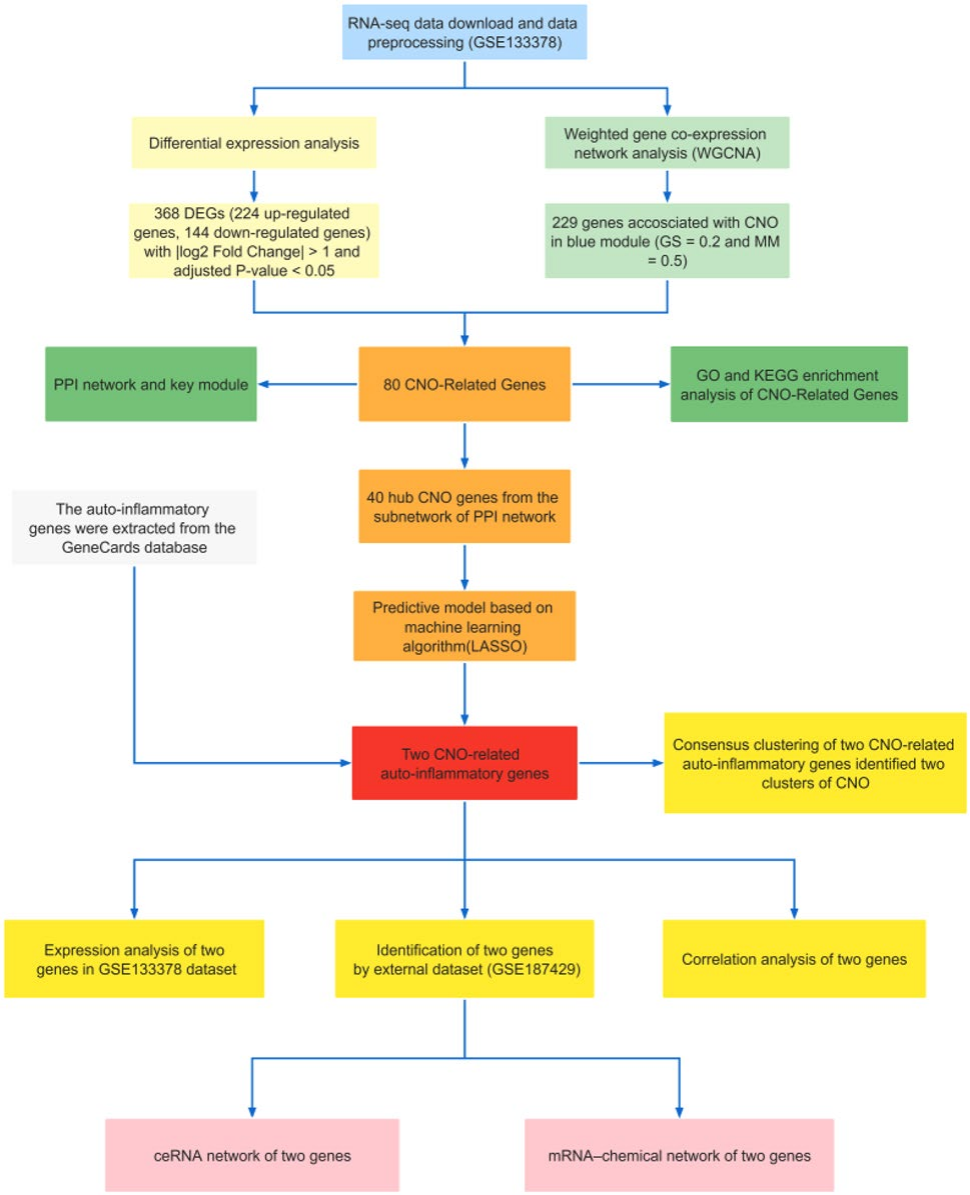
Flowchart of data analysis in this study. This included RNA-seq data download, differential expression analysis, and WGCNA analysis. Followed by the construction of PPI network and enrichment analysis, building machine learning models, screening of CNO-related auto-inflammatory genes in conjunction with the GeneCards database and construction of the ceRNA network, and finally validation with external datasets.

The search term “Chronic nonbacterial osteomyelitis AND human” was applied for dataset retrieval. Only two eligible RNA-seq datasets (GSE133378 and GSE187429) in GEO (https://www.ncbi.nlm.nih.gov/geo/) were selected and downloaded by using the “GEOquery” package of R software (version 4.1.2, http://r-project.org/) for subsequent bioinformatics analysis. The raw expression files of two microarray datasets were normalized with the “limma” package and “vst” function. Detailed information on the GEO datasets is listed in Table 1. The flow diagram of the study is shown in Fig. 1.

**Table 1:** Dataset details.

Dataset	Platform	Count	CNO	Control	Other
GSE133378	GPL18573 Illumina Nextseq 500 (*Homo sapiens*)	476	12	148	310
GSE80178	GPL24676 Illumina Novaseq 6000 (*Homo sapiens*)	20	11	9	–

### Weighted gene coexpression network analysis

The gene co-expression network was constructed with an R package termed “weighted gene coexpression network analysis (WGCNA)”^18^. First, the gene expression matrix is loaded in the R software to check for missing values and identify outliers. We construct a scale-free network to select a soft threshold value, which is considered to be the parameter cutoff-value for the construction of the adjacency and topology matrices. Second, the block module function and module division analysis were performed to identify gene co-expression modules. Each module was correlated with CNO and the most relevant modules were filtered according to Pearson correlation analysis coefficients. The genes in these modules were considered as CNO-related module genes.

### Identification of DEGs and CNO-related genes

It may be possible to clarify potential regulatory mechanisms for disease occurrence by identifying DEGs in different sample states and investigating their functions and interactions. First, the expression matrix data and grouping data were obtained by using R language based on the expression matrices of GSE133378 and series matrix files. Subsequently, DEGs between CNO and normal tissues were identified with the “DESeq2” package^19^. The threshold of |log2 fold change|> 1 and adjusted *P* value < 0.05 was set, and the genes satisfying these criteria were identified as DEGs. Finally, those genes that were found to belong to both the DEGs and the set of CNO-related module genes were considered genes related to CNO.

### GO and KEGG pathway enrichment analysis of the CNO-related genes

Gene Ontology (GO) and Kyoto Encyclopedia of Genes and Genomes (KEGG) pathway enrichment analyses were performed using the enrichGO and enrichKEGG functions of the “clusterProfiler” package, respectively. A *P* value < 0.05 was considered statistically significant. GO analysis consists of three main sets of terms, biological processes (BP), molecular functions (MF), and cellular components (CC). The results of the enrichment analysis were visualized by using the “ggplot2” package.

### PPI network construction and module analysis

The protein‒protein interaction (PPI) network of the proteins encoded by CNO-related genes was constructed with Search Tool for the Retrieval of Interacting Genes (STRING) (https://string-db.org/), with an interaction score of 0.15 as the threshold. Cytoscape, an application for visualizing molecular interaction networks, was used to construct the PPI network and identify the hub CNO genes based on the network file from STRING. The most important module was drawn by Cytoscape plugin molecular complex detection (MCODE) with the default settings: degree cut-off = 2, node score cut-off = 0.2, k-core = 2, and max depth = 100.

### Identifying and validating autoinflammatory genes

According to the R package “glmnet,” the least absolute shrinkage and selection operator (LASSO) machine learning model was applied to the hub CNO genes obtained from a subnetwork of the PPI network based on CytoHubba (Cytoscape plugin) to screen for key genes. The set of autoinflammatory genes (Supplementary Table 2) was obtained from the GeneCards database^20^ (GeneCards—Human Genes | Gene Database | Gene Search), which is a searchable, integrative database that provides comprehensive, user-friendly information on all annotated and predicted human genes. The autoinflammatory genes were intersected with the key genes, and the intersecting genes were recognized as autoinflammatory genes associated with CNO. The value of the prediction model and these genes as markers related to the progression of CNO was assessed in GSE187429 as an external validation dataset by applying the “pROC” package. The differential expression of the obtained genes in different groups of samples was calculated by the “RColorBrewer” package.

### Autoinflammatory genes of analysis

Unsupervised consensus clustering is a broadly useful method for discovering biological features that has been used in a variety of studies^21^. We applied the consistent clustering algorithm to determine the clustering number of samples. Small clusters were not allowed, and the cluster number k was set from 2 to 8. The cumulative distribution function (CDF) and area under the CDF curve were used to confirm the optimal cluster number. Then, we performed PCA to verify the clustering results. Ultimately, we used NetworkAnalyst^22^ (NetworkAnalyst) to construct a gene regulation network consisting of the lncRNA‒miRNA–mRNA interactions and a protein–chemical interaction network.

## Results

### WGCNA and identification of core modules

**Figure 2:**
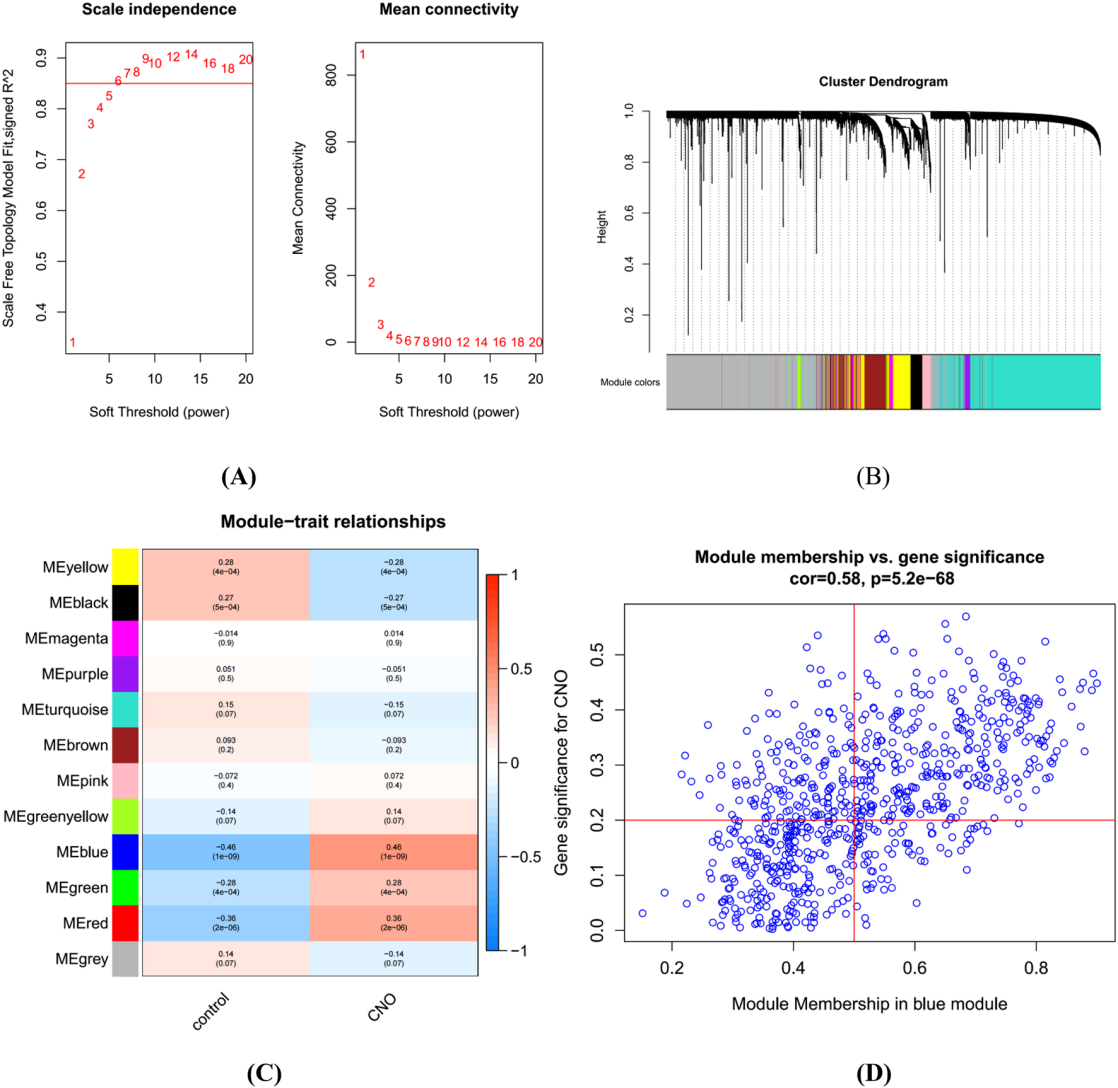
Weighted gene coexpression network analysis (WGCNA). (**A**) Network topology analysis under various soft-threshold powers. Left: Analysis of the scale-free index for various soft-threshold powers (β). Right: Analysis of the mean connectivity for various soft-threshold powers. (**B**) Identification of coexpressed gene modules. The branches of the dendrogram cluster into eleven modules, and each module is labelled in a unique colour. (**C**) A heatmap showing the correlation between each module eigengene and phenotype. (**D**) The relevance of members in the blue module to CNO. GS: Correlation of all gene expression profiles with module genes. MM: Absolute values of correlations between genes and phenotypic traits.

The soft threshold (R^2^ = 0.85) for construction of the scale-free network was set at six (Fig. 2A). The adjacency matrix converted from the expression matrix was transformed into a topological matrix, and gene clustering was conducted based on the average linkage hierarchical clustering method. We identified eleven signature modules labelled with different colours; the genes within each module can represent the overall gene expression level of each module (Fig. 2B). The module with the highest correlation with CNO was blue, r = 0.46, P = 1e − 09 (Fig. 2C). The correlation between genes in the blue module and CNO genes was cor = 0.58, p = 5.2e − 68; a total of 229 genes (Supplementary Table 1) most associated with CNO were screened from this module based on Gene significance (GS) = 0.2 and Module Membership (MM) = 0.5 (Fig. 2D).

### Identification of DEGs and CNO-related genes

**Figure 3:**
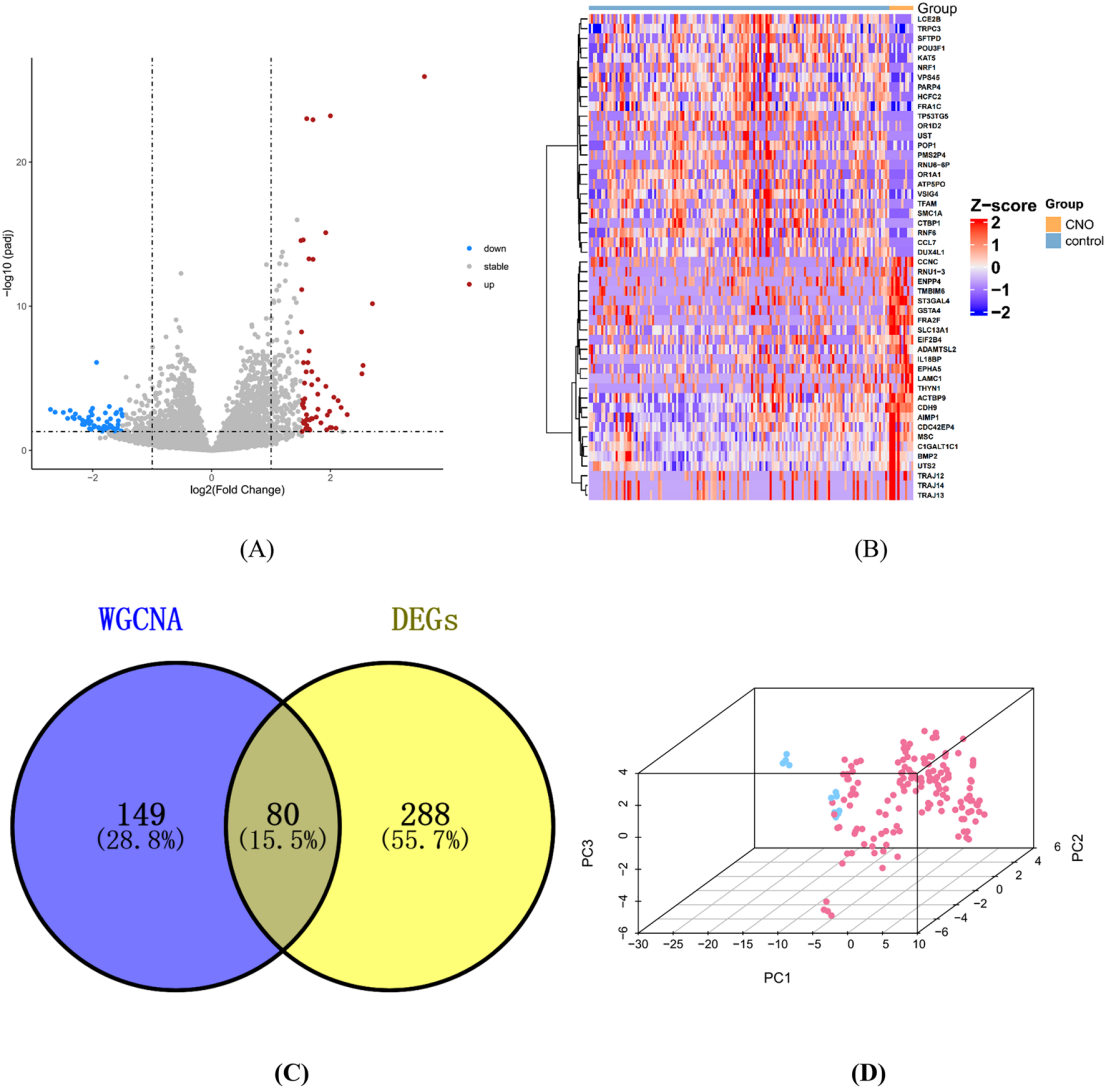
Identification of DEGs and CNO-related genes. (**A**) Volcano plot: The red points represent upregulated genes, and blue points represent downregulated genes. Genes with no significant difference are shown in grey. The threshold for differential expression was set as |log2 fold change|> 2 and adjusted P value < 0.05. (**B**) Heatmap: The heatmap of the top 25 upregulated genes and the top 25 downregulated genes; blue indicates relatively low expression, and red indicates relatively high expression. (**C**) Venn diagram: The DEGs and CNO-related module genes showed an overlap of 80 genes. (**D**) PCA: Dimension reduction analysis of the 80 genes.

In total, 368 DEGs, consisting of 224 upregulated and 144 downregulated genes, were identified by the “DESeq2” package based on the threshold value of |log2 fold change|> 1 and adjusted p value < 0.05. The volcano plot of all DEGs and the heatmap of the top 25 upregulated genes and the top 25 downregulated genes were visualized (Fig. 3A,B). The details of the DEGs are shown in Supplementary Table 1. Finally, eighty genes belonging jointly to these two groups of genes were considered to be associated with CNO (Fig. 3C). In addition, as shown in Fig. 3D, the results of the principal component analysis (PCA) based on the “ComplexHeatmap” package indicated that eighty genes could effectively distinguish between normal samples and CNO.

### GO and KEGG analysis

**Figure 4:**
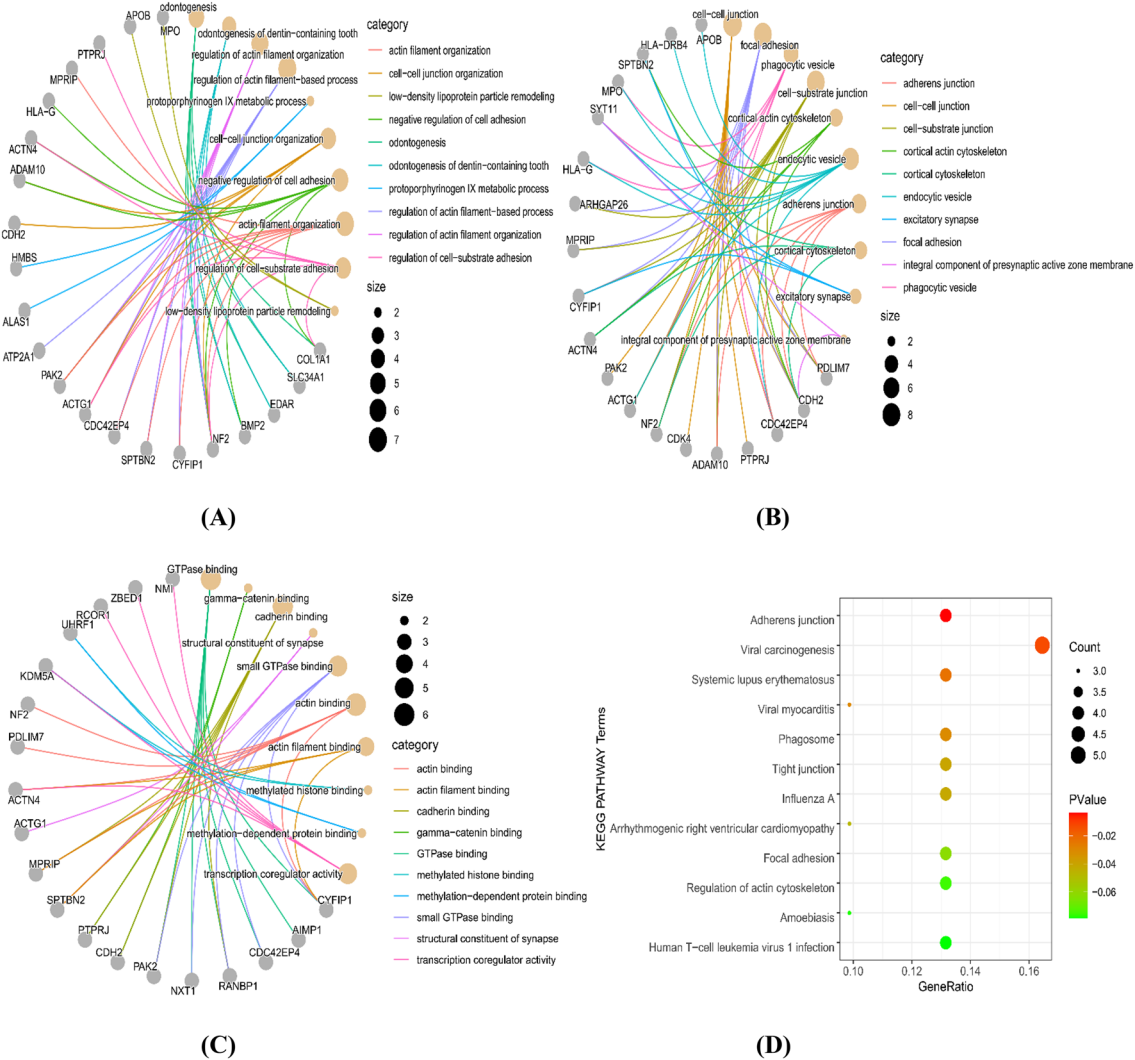
GO and KEGG enrichment analyses in CNO. (**A**–**C**) The top 10 GO terms in the biological process (BP), cellular component (CC) and molecular function (MF) categories. (**D**) KEGG pathway analysis of CNO-related genes.

The biological function and pathway analyses were conducted using the R package clusterProfiler. Enrichment of the three GO categories (BP, CC, MF) within the DEGs (Fig. 4A,B,C) indicated that they were mainly associated with regulation of actin filament organization, cell‒cell junction organization, gamma-catenin binding, and actin binding (Supplementary Table 3). As shown in Fig. 4D, the enriched pathways were mainly involved in adherens junction (hsa04520), viral carcinogenesis (hsa05203), systemic lupus erythematosus (hsa05322), viral myocarditis (hsa05416), and phagosome (hsa04145) (Supplementary Table 3)^23^.

### PPI network and module analysis

**Figure 5:**
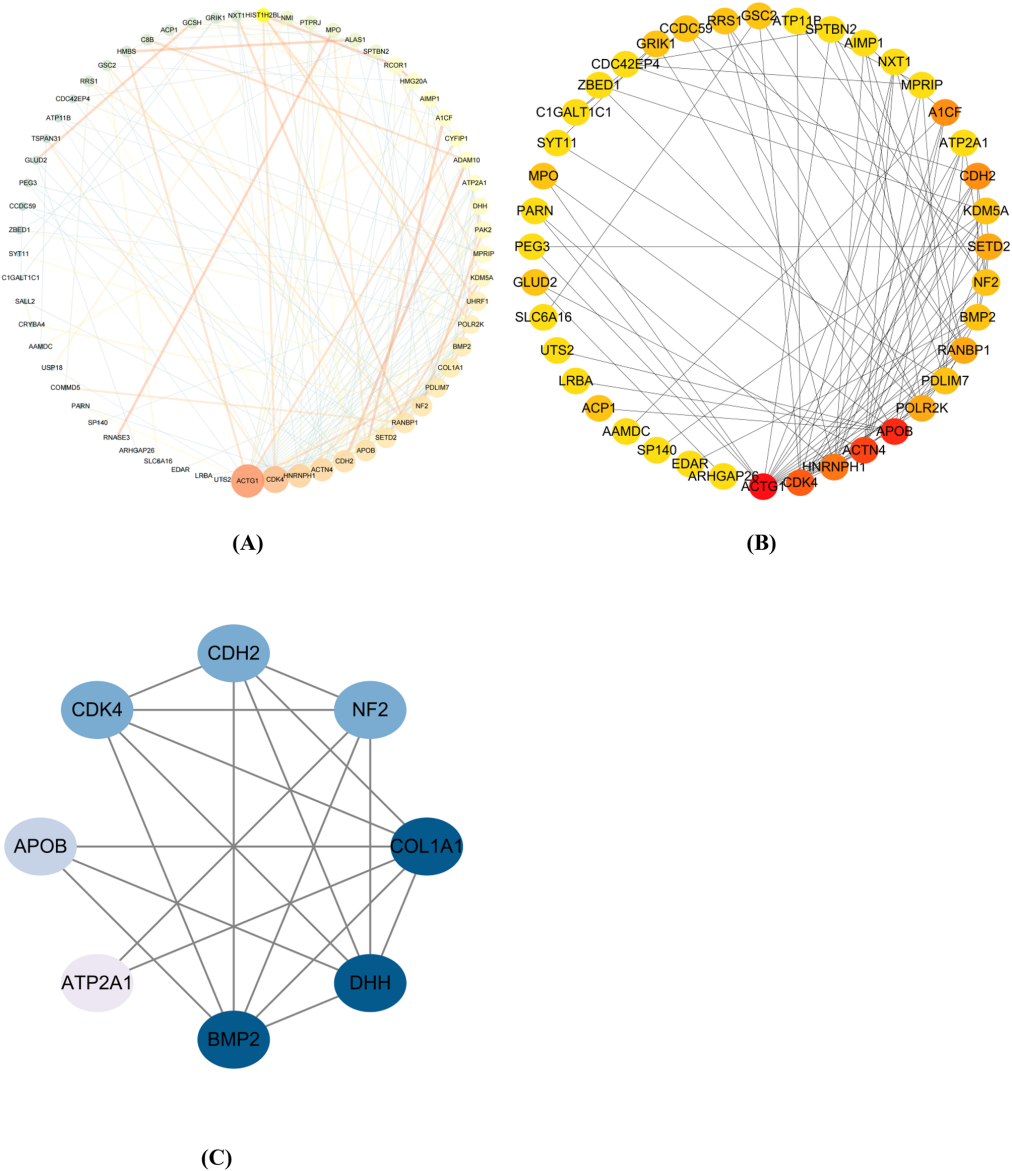
PPI network and module analysis. (**A**) PPI network of CNO-related genes. (**B**) The subnetwork of 40 hub CNO genes. (**C**) The key module of the PPI network.

The eighty identified CNO-related genes were submitted to the STRING database, and we acquired PPI raw files, which provide valuable information about molecular mechanisms in physiological and pathological processes. After the isolated nodes were removed by Cytoscape 3.7.1, the PPI network of CNO-related genes was generated (Fig. 5A). A subnetwork of the PPI network containing 40 hub CNO genes was identified by the bottleneck algorithm with the cytoHubba plugin (Fig. 5B). Then, the key module containing 8 genes was identified via the MCODE plugin (Fig. 5C).

### Identification and validation of autoinflammatory genes

**Figure 6:**
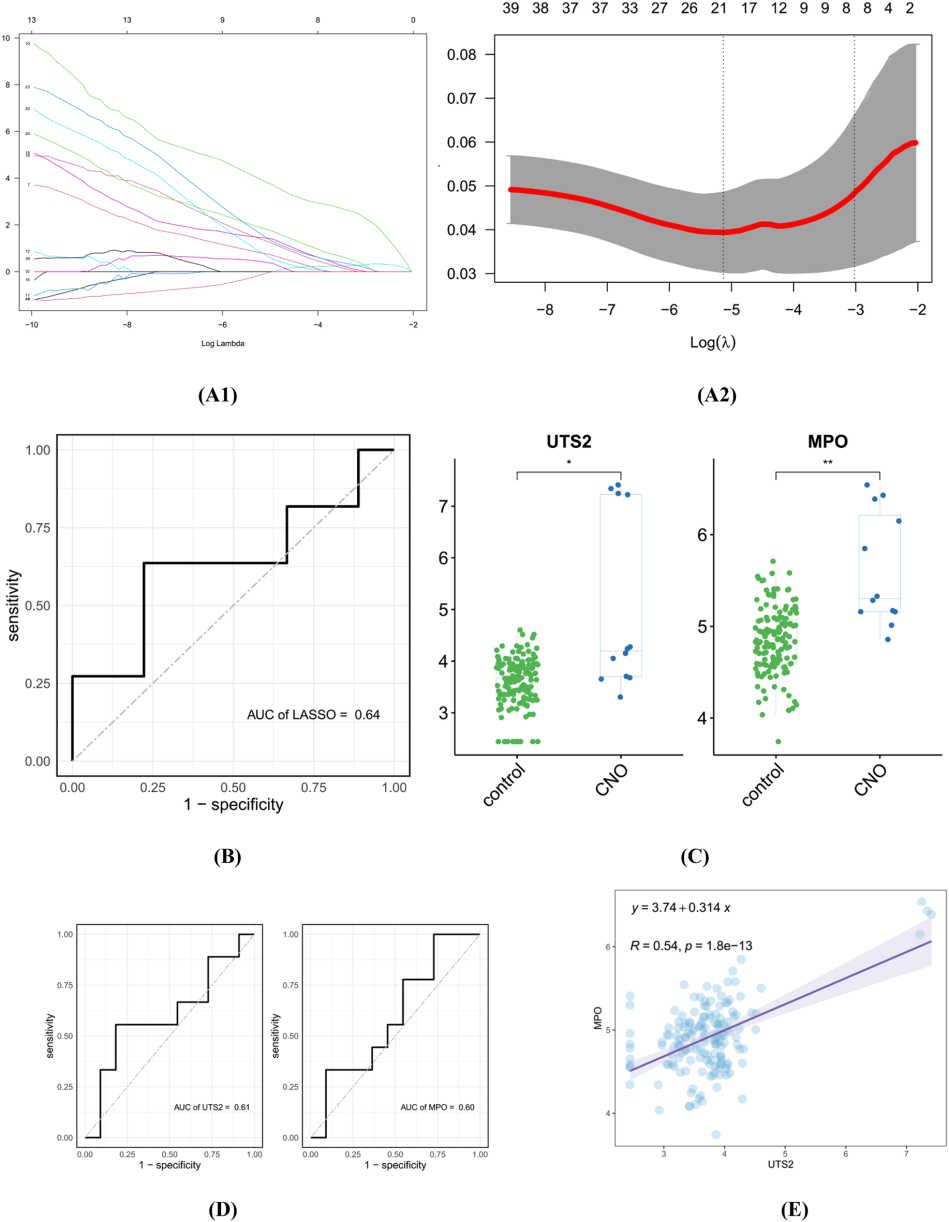
Identification and validation of autoinflammatory genes in CNO. (**A1**,**A2**) Twenty key genes obtained by applying the LASSO algorithm based on the minimum lambda. (**B**) Validation of the predictive model in external datasets. (**C**) Expression of the two CNO-related autoinflammatory genes in CNO samples and control samples; the differences were statistically significant (*p < 0.05; **p < 0.01). (**D**) Validation of the two CNO-related autoinflammatory genes in external datasets. (**E**) Correlation of the two CNO-related autoinflammatory genes; the correlation was statistically significant (p < 0.05).

We identified twenty key genes by applying the validated machine learning algorithms (LASSO) to the forty hub CNO genes (Fig. 6A1,A2). We used GSE187429 as an external dataset to evaluate the efficiency of the supervised machine learning algorithms using ROC curves (Fig. 6B). The AUC value of the LASSO model was 0.64, and we considered it the optimal CNO prediction model. According to the set of autoinflammatory genes in the GeneCards database, there were two genes that were associated with autoinflammation among the twenty key genes related to CNO. In the GSE133378 dataset, the two genes were both highly expressed in CNO samples relative to normal samples (Fig. 6C). Finally, ROC curves were plotted for the external validation set data (GSE187429) to verify the potential value of the two genes as early diagnostic markers or therapeutic targets for CNO patients. The AUC values of UTS2 and MPO were 0.61 and 0.60, respectively, which were both greater than or equal to 0.60, and these genes were therefore identified as CNO-related hub genes (Fig. 6D). In addition, Fig. 6E illustrates that there was a positive correlation between the two genes.

### Autoinflammatory genes identified by CNO analysis

**Figure 7:**
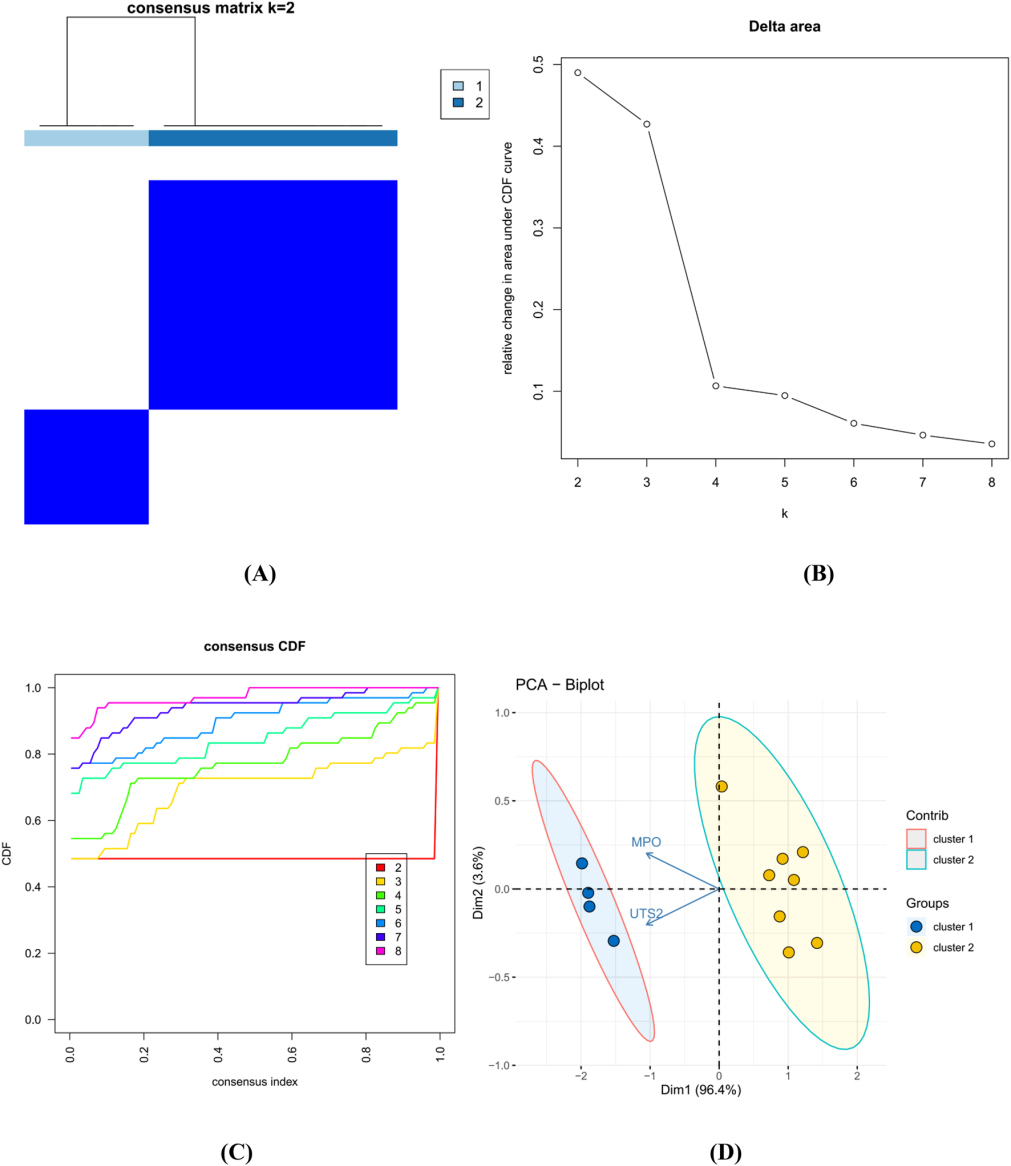
Identification of consensus clusters by the two CNO-related autoinflammatory genes. (**A**) Consensus matrix of autoinflammatory genes when k = 2. (**B**) Relative change in the area under the cumulative distribution function (CDF) curve for k values from 2 to 8. (**C**) CDF plot when the k value ranges from 2 to 8. (**D**) PCA of the two autoinflammatory genes in the CNO samples.

**Figure 8:**
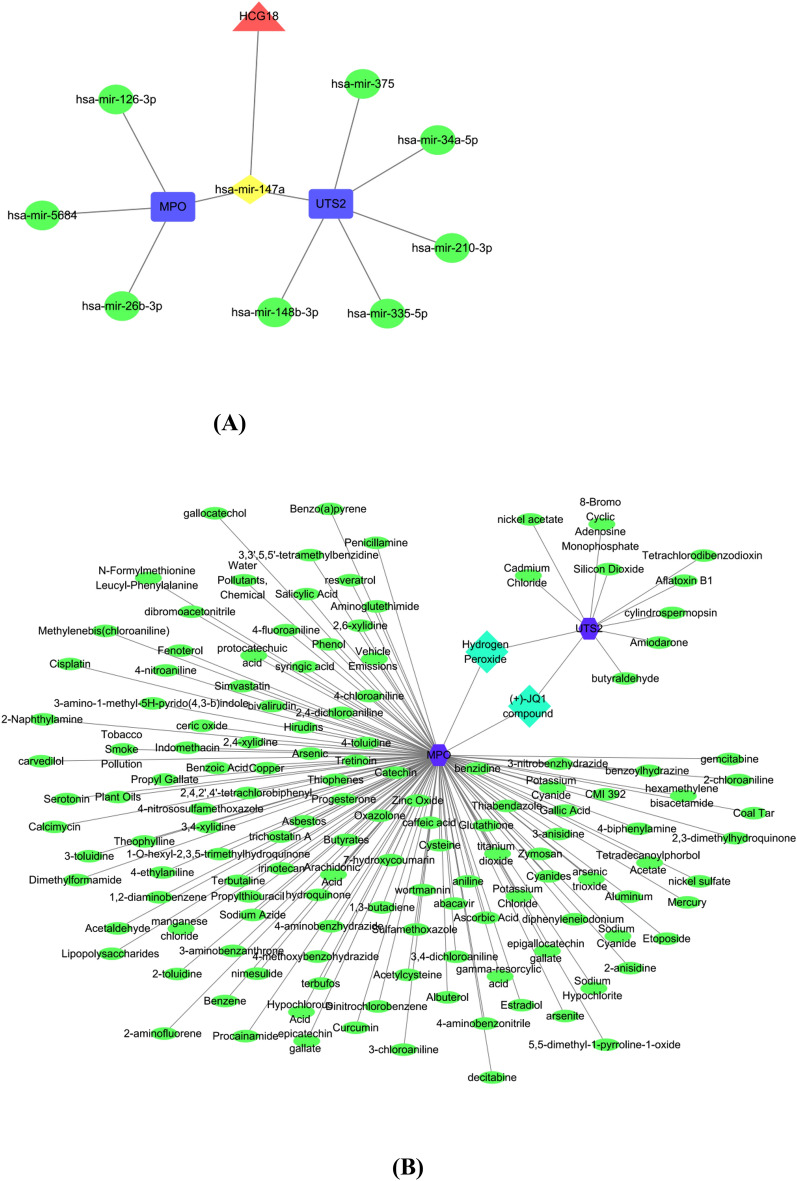
Network analysis of the two CNO-related autoinflammatory genes. (**A**) ceRNA network: An lncRNA is shown in red, miRNAs are shown in green, two mRNAs are shown in blue, and the miRNA common to the two mRNAs is shown in yellow. (**B**) mRNA–chemical interactive network. The two mRNAs are shown in blue, small molecule compounds are shown in green, and the common small molecule compounds common to the two mRNAs are shown in light blue.

Based on the two CNO-related autoinflammatory genes identified above, CNO subtype analysis was performed. According to Fig. 7B and C, k values of 2 or 3 would be acceptable; however, after dividing the samples into 3 groups, some data could not be well clustered; therefore, we elected to separate our data into 2 groups. The data could be well clustered when k = 2 (k: clustering variable), as shown in Fig. 7B and C. The matrix shown in Fig. 7A represents the consensus for k = 2 and indicates a well-defined two-block structure. As shown in Fig. 7D, two CNO-related autoinflammatory genes could distinguish cluster 1 from cluster 2 and were expressed differentially between the two clusters. We concluded that grouping by CNO-related autoinflammatory gene expression was appropriate (k = 2). To explore the upstream targets of these two CNO-related autoinflammatory genes, we used the online tool NetworkAnalyst to predict the miRNAs associated with the two genes^24^. The starBase database (https://starbase.sysu.edu.cn/) was employed with both low-stringency (≥ 1) and high-stringency (≥ 3) criteria to predict lncRNAs based on hsa-mir-147a belonging to two genes in common, as well as to construct a ceRNA network (Fig. 8A). As the key genes may carry out the dominant role, we also continued to explore the interaction between hub genes and small molecule compounds and provided references for possible therapeutic drugs. We used the online tool NetworkAnalyst to predict compounds targeting these two genes and to construct an mRNA–chemical network (Fig. 8B).

## Discussion

CNO is a rare autoinflammatory disease whose pathophysiological mechanisms are unknown, resulting in a high rate of misdiagnosis and a lack of effective therapeutic agents. Therefore, it is important to understand the precise molecular mechanisms of CNO development to develop an effective diagnosis and find alternative treatment strategies.

In this study, GSE133378 gene expression profiles were downloaded and used for WGCNA and differential expression analysis. Ultimately, eighty genes closely related to CNO were identified according to the specific threshold set. Enrichment analysis showed that these genes were mainly associated with cell junctions, adhesion, and protein binding, including regulation of actin filament organization, cell‒cell junction organization, gamma-catenin binding, and actin binding. These annotation results provide valuable clues to the molecular interactions in the development of CNO. Indeed, cell adhesion and attachment play a crucial role in the inflammatory response. For example, adhesion and transendothelial migration of neutrophils can promote the inflammatory process^25^. In addition, leukocytes exfiltrate through the circulatory system to the site of inflammation, where they adhere to the vessel wall to control inflammation^26^. Actin is a globular protein that polymerizes to form cytoskeletal microfilaments, the main component of which is the polar linear polymer actin filament that forms from abundant cytoplasmic actin protein^27^. Previously, it was generally believed that the most important function of actin was to perform muscle contraction in concert with troponin. However, a growing body of evidence suggests that dysregulation of the actin cytoskeleton is associated with autoinflammatory diseases and primary immunodeficiency^28^. The field of autoinflammation describes how the actin cytoskeleton facilitates the autoinflammatory process and the spectrum of conditions characterized by the coexistence of inflammatory, autoimmune and defective immune responses. In fact, the complex dysregulation of actin remodelling is an example of an autoinflammatory disease combined with immunodeficiency^29^. We will follow up with more in-depth research and analysis in this direction. As shown in the KEGG pathway analysis, CNO-related genes were enriched in pathways such as “viral carcinogenesis”, “systemic lupus erythematosus” and “viral myocarditis”. This suggests that genes or pathways related to viral carcinogenesis, systemic lupus erythematosus and viral myocarditis might have important roles in the pathophysiology of CNO.

It has been reported that genes such as LPIN2, IL1RN and FBLIM are associated with the pathogenesis of CNO, but mutations in these genes are present in only a very few cases of CNO^30^. Thus, the role of genetics in the pathogenesis of CNO is unclear, and finding additional genes that are closely associated with CNO pathogenesis has become important. We identified two autoinflammatory genes (UTS2 and MPO) associated with CNO by combining an autoinflammatory response-related gene set with the LASSO machine learning algorithm. Both genes were highly expressed in CNO patients. The AUC values under the ROC curves for UTS2 and MPO were 0.61 and 0.60, respectively, indicating that both genes are closely associated with autoinflammation. The urotensin II (UTS2) gene is present on human chromosome 1p36-p32 and produces an 11 amino acid peptide with multiple vasoprotective and vasculopathic effects^31^. UTS2 is commonly expressed in the renal, cardiovascular, and endocrine systems; recent studies have shown that UTS2 is widely distributed in the central nervous system (CNS) and peripheral tissues^32^. Although a large number of studies have been conducted on the association of the UTS2 gene with various diseases, an association with CNO has not been reported. In a UTS2 rs228648 (p.Thr21Met) variant analysis, Asmaa et al. revealed that the Thr/Thr genotype and Thr allele were significantly associated with an increased risk of developing Behçet’s disease, a chronic autoimmune inflammatory disease^33^. This result provides a solid theoretical basis for our study of the relationship between UTS2 and the autoinflammatory disease CNO. The myeloperoxidase (MPO) gene encodes myeloid oxidase, a heme-containing peroxidase that is highly expressed in a variety of inflammatory cells, including neutrophils, activated microglia, monocytes/macrophages, astrocytes and neurons^34^. In recent studies, it was found that functional mutations in MPO, a regulator of neutrophil-associated inflammation in the skin, can induce pustular psoriasis^35^. The common presence of *Cutibacterium acnes* within bone biopsy samples in adult patients diagnosed with synovitis, acne, pustulosis, hyperostosis, osteitis (SAPHO) syndrome, thought to be a subtype of CNO that occurs mostly in adults, suggested that this bacterial species may be associated with the disease^36^. Combined with the ROC curve results of MPO in this study (AUC = 0.60), this observation gives us reason to believe that MPO plays a specific role in the pathogenesis of CNO.

To explore the profound mechanisms by which UTS2/MPO affects the pathogenesis of CNO, we constructed a ceRNA network through a network database and defined the HCG18/has-mir-147a/UTS2/MPO axis. Multiple studies have indicated that HCG18 is involved in a variety of human nononcological diseases, including diabetic peripheral neuropathy and intervertebral disc degeneration^37,38^. Moreover, it is clear that HCG18 is dysregulated in many tumours and can predict the prognosis of certain tumours. However, the role of HCG18 in CNO has not been reported. The upregulation of HCG18 promotes macrophage enrichment, which further regulates the inflammatory response and pro-inflammatory cytokine release in macrophages^37^. This inflammation-related response caused by HCG18 is highly likely to have a strong correlation with the pathogenesis of CNO. Therefore, on the basis of the HCG18/has-mir-147a/UTS2/MPO axis, we hypothesize that the lncRNA HCG18 can act as an upstream regulator and exert a specific regulatory effect on UTS2/MPO target genes by interacting with hsa-mir-147a.

The initial identification of these molecular markers in this study lays the foundation for further studies on the pathogenesis of CNO, but certain limitations remain. The rarity of the disease makes the number of samples available in public databases limited. Therefore, considering the potential analysis bias, we will increase the sample size in our subsequent work and combine in vitro and in vivo experiments to more deeply explore the role of the identified autoinflammation-related genes in the development of CNO and as possible targets for treatment and related mechanisms.

## Conclusion

We identified two hub genes (UTS2 and MPO) that are closely associated with auto-inflammatory in CNO and can differentiate CNO patients from controls, and are thus potential auto-inflammatory-related biomarkers for disease diagnosis and therapeutic monitoring. In addition, our findings suggest that the lncRNA HCG18 can act as an upstream regulator and exert a specific regulatory effect on the UTS2/MPO target genes by interacting with hsa-mir-147a. In the future, these molecular markers deserve further study in follow-up and require additional datasets and further experimental validation at the cellular or specimen level.

## Supplementary Information

Supplementary Legends.

Supplementary Table 1.

Supplementary Table 2.

Supplementary Table 3.

## Data Availability

The datasets analysed during the current study are available in the Gene Expression Omnibus (GEO) and GeneCards repository, [GSE133378, GSE187429].

## Abbreviations

CNO: Chronic nonbacterial osteomyelitis
GEO: Gene expression omnibus
WGCNA: Weighted gene co-expression network analysis
DEGs: Differentially expressed genes
ROC: Receiver operating characteristic
GO: Gene ontologyand
KEGG: Kyoto encyclopedia of genes and genomes
BP: Biological processes
MF: Molecular functions
CC: Cellular components
PPI: Protein–protein interaction
CDF: Cumulative distribution function
PCA: Principal component analysis
SAPHO: Synovitis, acne, pustulosis, hyperostosis, osteitis

## References

[1] Schnabel A, Range U, Hahn G, Siepmann T, Berner R, Hedrich CM (2016). Unexpectedly high incidences of chronic non-bacterial as compared to bacterial osteomyelitis in children. Rheumatol. Int..

[2] Björkstén B, Gustavson KH, Eriksson B, Lindholm A, Nordström S (1978). Chronic recurrent multifocal osteomyelitis and pustulosis palmoplantaris. J. Pediatr..

[3] Wipff J, Costantino F, Lemelle I, Pajot C, Duquesne A, Lorrot M (2015). A large national cohort of French patients with chronic recurrent multifocal osteitis. Arthritis Rheumatol..

[4] Grote V, Silier CCG, Voit AM, Jansson AF (2017). Bacterial osteomyelitis or nonbacterial osteitis in children: A study involving the German surveillance unit for rare diseases in childhood. Pediatr. Infect. Dis. J..

[5] Hofmann SR, Morbach H, Schwarz T, Rösen-Wolff A, Girschick HJ, Hedrich CM (2012). Attenuated TLR4/MAPK signaling in monocytes from patients with CRMO results in impaired IL-10 expression. Clin. Immunol..

[6] Scianaro R, Insalaco A, Bracci Laudiero L, De Vito R, Pezzullo M, Teti A (2014). Deregulation of the IL-1β axis in chronic recurrent multifocal osteomyelitis. Pediatr. Rheumatol. Online J..

[7] Koryllou A, Mejbri M, Theodoropoulou K, Hofer M, Carlomagno R (2021). Chronic nonbacterial osteomyelitis in children. Children.

[8] Cox AJ, Darbro BW, Laxer RM, Velez G, Bing X, Finer AL (2017). Recessive coding and regulatory mutations in FBLIM1 underlie the pathogenesis of chronic recurrent multifocal osteomyelitis (CRMO). PLoS One.

[9] Abe K, Cox A, Takamatsu N, Velez G, Laxer RM, Tse SML (2019). Gain-of-function mutations in a member of the Src family kinases cause autoinflammatory bone disease in mice and humans. Proc. Natl. Acad. Sci. U. S. A..

[10] Zhao Y, Ferguson PJ (2018). Chronic nonbacterial osteomyelitis and chronic recurrent multifocal osteomyelitis in children. Pediatr. Clin. N. Am..

[11] Hofmann SR, Kapplusch F, Girschick HJ, Morbach H, Pablik J, Ferguson PJ (2017). Chronic recurrent multifocal osteomyelitis (CRMO): Presentation, pathogenesis, and treatment. Curr. Osteoporos. Rep..

[12] Winters R, Tatum SA (2014). Chronic nonbacterial osteomyelitis. Curr. Opin. Otolaryngol. Head Neck Surg..

[13] Costa-Reis P, Sullivan KE (2013). Chronic recurrent multifocal osteomyelitis. J. Clin. Immunol..

[14] Frésard L, Smail C, Ferraro NM, Teran NA, Li X, Smith KS (2019). Identification of rare-disease genes using blood transcriptome sequencing and large control cohorts. Nat. Med..

[15] Mohammadi P, Castel SE, Cummings BB, Einson J, Sousa C, Hoffman P (2019). Genetic regulatory variation in populations informs transcriptome analysis in rare disease. Science.

[16] Azevedo T, Dimitri GM, Lió P, Gamazon ER (2021). Multilayer modelling of the human transcriptome and biological mechanisms of complex diseases and traits. NPJ Syst. Biol. Appl..

[17] Zhang W, Dong R, Diao S, Du J, Fan Z, Wang F (2017). Differential long noncoding RNA/mRNA expression profiling and functional network analysis during osteogenic differentiation of human bone marrow mesenchymal stem cells. Stem Cell Res. Ther..

[18] van Eijk KR, de Jong S, Boks MPM, Langeveld T, Colas F, Veldink JH (2012). Genetic analysis of DNA methylation and gene expression levels in whole blood of healthy human subjects. BMC Genom..

[19] Zago E, Dal Molin A, Dimitri GM, Xumerle L, Pirazzini C, Bacalini MG (2022). Early downregulation of hsa-miR-144-3p in serum from drug-naïve Parkinson’s disease patients. Sci. Rep..

[20] Safran M, Rosen N, Twik M, BarShir R, Stein TI, Dahary D, Abugessaisa I, Kasukawa T (2021). The GeneCards suite. Practical Guide to Life Science Databases.

[21] Spiga O, Cicaloni V, Dimitri GM, Pettini F, Braconi D, Bernini A (2021). Machine learning application for patient stratification and phenotype/genotype investigation in a rare disease. Brief Bioinform..

[22] Zhou G, Soufan O, Ewald J, Hancock REW, Basu N, Xia J (2019). NetworkAnalyst 3.0: A visual analytics platform for comprehensive gene expression profiling and meta-analysis. Nucleic Acids Res..

[23] Kanehisa M, Furumichi M, Sato Y, Ishiguro-Watanabe M, Tanabe M (2021). KEGG: Integrating viruses and cellular organisms. Nucleic Acids Res..

[24] Suzuki HI, Young RA, Sharp PA (2017). Super-enhancer-mediated RNA processing revealed by integrative MicroRNA network analysis. Cell.

[25] Winkler MJ, Müller P, Sharifi AM, Wobst J, Winter H, Mokry M (2020). Functional investigation of the coronary artery disease gene SVEP1. Basic Res. Cardiol..

[26] Paddillaya N, Mishra A, Kondaiah P, Pullarkat P, Menon GI, Gundiah N (2019). Biophysics of cell-substrate interactions under shear. Front. Cell Dev. Biol..

[27] Svitkina T (2018). The actin cytoskeleton and actin-based motility. Cold Spring Harb. Perspect. Biol..

[28] Papa R, Picco P, Gattorno M (2020). The expanding pathways of autoinflammation: A lesson from the first 100 genes related to autoinflammatory manifestations. Adv. Protein Chem. Struct. Biol..

[29] Papa R, Penco F, Volpi S, Gattorno M (2020). Actin remodeling defects leading to autoinflammation and immune dysregulation. Front. Immunol..

[30] Buch K, Thuesen ACB, Brøns C, Schwarz P (2019). Chronic non-bacterial osteomyelitis: A review. Calcif. Tissue Int..

[31] Russell FD (2008). Urotensin II in cardiovascular regulation. Vasc. Health Risk Manag..

[32] Cui L, Lv C, Zhang J, Li J, Wang Y (2021). Characterization of four urotensin II receptors (UTS2Rs) in chickens. Peptides.

[33] Kamal A, Elgengehy FT, Elawady Z, Fawzy NA, El Sisi O (2022). Role of miR-146a rs2910164 and UTS2 rs228648 genetic variants in Behcet’s disease. Immunol. Investig..

[34] Chen S, Chen H, Du Q, Shen J (2020). Targeting myeloperoxidase (MPO) mediated oxidative stress and inflammation for reducing brain ischemia injury: Potential application of natural compounds. Front. Physiol..

[35] Haskamp S, Bruns H, Hahn M, Hoffmann M, Gregor A, Löhr S (2020). Myeloperoxidase modulates inflammation in generalized pustular psoriasis and additional rare pustular skin diseases. Am. J. Hum. Genet..

[36] Nuruzzaman F, Zhao Y, Ferguson PJ (2021). Chronic nonbacterial osteomyelitis: Insights into pathogenesis, assessment, and treatment. Rheum. Dis. Clin. N. Am..

[37] Xi Y, Jiang T, Wang W, Yu J, Wang Y, Wu X (2017). Long non-coding HCG18 promotes intervertebral disc degeneration by sponging miR-146a-5p and regulating TRAF6 expression. Sci. Rep..

[38] Zhu Y, Zhao J, Tan L, Lin S, Long M, Peng X (2021). LncRNA-HCG18 regulates the viability, apoptosis, migration, invasion and epithelial-mesenchymal transition of papillary thyroid cancer cells via regulating the miR-106a-5p/PPP2R2A axis. Pathol. Res. Pract..

