# Supplementary material for: Transcriptome analysis based on machine learning reveals a role for autoinflammatory genes of chronic nonbacterial osteomyelitis (CNO)
Source: Sci Rep. 2023 Apr 21;13:6514. doi: 10.1038/s41598-023-33759-y (PMC10121556; doi:10.1038/s41598-023-33759-y)
Supplement: Supplementary file 1 — Supplementary Legends. [file 41598_2023_33759_MOESM1_ESM.pdf]

Supplementary Materials

Supplementary Table 1: The details of the DEGs, WGCNA and the the intersection of DEGs and WGCNA.

Supplementary Table 2: The detailes of GeneCards, 40 hub CNO genes, 20 key genes and two auto-inflammatory genes of CNO.

Supplementary Table 3: The detailed results of the GO enrichment analyses and KEGG enrichment pathway analyses.
